# Insulin resistance in vitamin D-deficient mice is alleviated by n-acetylcysteine

**DOI:** 10.18632/oncotarget.18793

**Published:** 2017-06-28

**Authors:** Zhao-Hui Cui, Qi Yuan, Li Mao, Feng-Li Chen, Feng Ji, Sha Tao

**Affiliations:** ^1^ Department of Endocrinology, Huai'an First People's Hospital, Nanjing Medical University, Huai'an, China; ^2^ Clinical Laboratory, Huai'an First People's Hospital, Nanjing Medical University, Huai'an, China; ^3^ Department of Orthopedics, Huai'an First People's Hospital, Nanjing Medical University, Huai'an, China

**Keywords:** vitamin D, insulin resistance, oxidative stress, N-acetylcysteine (NAC), inflammation

## Abstract

Vitamin D deficiency will lead to insulin resistance. In the current study, vitamin D3 1α-Hydroxylase [“1α(OH)ase”] knockout mice were generated to mimic vitamin D deficiency *in vivo*. As compared to the wild-type mice, the liver tissues of the knockout mice showed impaired insulin signaling, decreased glucose transporter 4 expression and increased reactive oxygen species production. Meanwhile, p53-p21 activation, apoptosis intensity and pro-inflammatory cytokines (IL-6, IL-1 and MIP-1α) level were significantly increased in the knockout mice livers. Significantly, such effects in the knockout mice were largely attenuated by supplement with anti-oxidant n-acetylcysteine (NAC). Remarkably, insulin resistance and metabolic abnormalities in the knockout mice were largely alleviated after treatment of NAC. Therefore, inhibition of oxidative stress by NAC alleviated insulin resistance in vitamin D-deficient mice. Oxidative stress could be the primary cause of insulin resistance by vitamin D deficiency.

## INTRODUCTION

One major cause and characteristic marker of type-II diabetes is insulin resistance [1–3]. It could be induced by various genetic abnormalities and/or environmental factors [1–3]. Literatures have proposed a link between vitamin D deficiency and insulin resistance [4]. Studies have shown that supplementation of active vitamin D3 might improve the insulin sensitivity [4]. Yet, the underlying mechanisms are largely undefined [4].

The abnormal production of reactive oxygen species (ROS) and oxidative stress could cause insulin resistance [5–7]. Existing evidences have shown that increased ROS level was associated with insulin resistance in patients with type-II diabetes [8–10]. Further, exogenous introduction of oxidative stress could lead to insulin resistance, indicating that oxidative stress is crucial in the development of insulin resistance [5, 7]. On the other hand, ROS scavenging would improve insulin sensitivity and glucose homeostasis in diabetic mice and patients [8–10].

The synthesis of vitamin D3 requires 25-Hydroxyvitamin D3 1α-Hydroxylase [“1α(OH)ase”] [11, 12]. In the current study, 1α(OH)ase knockout (“KO”) mice were generated to achieve vitamin D deficiency *in vivo*. Our results show that vitamin D deficiency causes insulin resistance probably by provoking oxidative stress in mice. N-acetylcysteine (NAC), a well-known anti-oxidant, restored insulin sensitivity in vitamin D deficiency mice.

## RESULTS

### Knockout of 1α(OH)ase leads to vitamin D deficiency in mice

**Figure 1:**
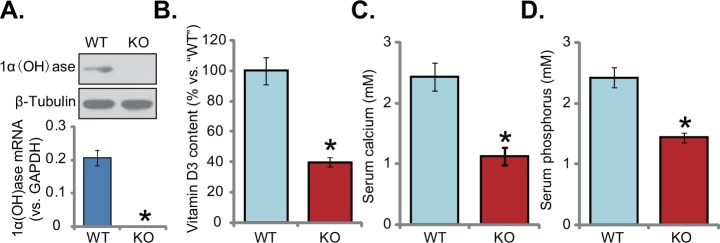
Knockout of 1α(OH)ase leads to vitamin D deficiency in mice The liver tissues of wild-type (“WT”) and 1α(OH)ase knockout (“KO”) mice were isolated at 21-day of age; Expression of 1α(OH)ase was tested by Western blotting assay and qRT-PCR assay **(A)**, Serum vitamin D3 **(B)**, calcium **(C)** and phosphorus **(D)** were also tested. Data were expressed as mean ± SE (n=5). * *p* <0.05 *vs*. “WT” mice.

Generation and characterization of 1α(OH)ase knockout (“KO”) mice were performed as described previously [13, 14]. Fresh liver tissues were isolated from both wild-type (“WT”) and KO mice. Western blotting assay results in Figure 1A (upper panel) confirmed that 1α(OH)ase protein was completely depleted in the liver tissues of KO mice. *1α(OH)ase mRNA* expression was also deleted in the KO mice (Figure 1A, lower panel). Importantly, serum vitamin D3 content was remarkably lower than that of WT mice (Figure 1B). Vitamin D3 content in the KO mice was around 30-40% of that in the WT mice (Figure 1B). Consequently, serum level of calcium (Figure 1C) and phosphorus (Figure 1D) were also significantly decreased in the KO mice. These results together confirm the phonotype of the KO mice, showing vitamin D deficiency.

### Supplement with NAC or 1,25(OH)_2_D_3_ rescues insulin signaling in 1α(OH)ase KO mice liver

**Figure 2:**
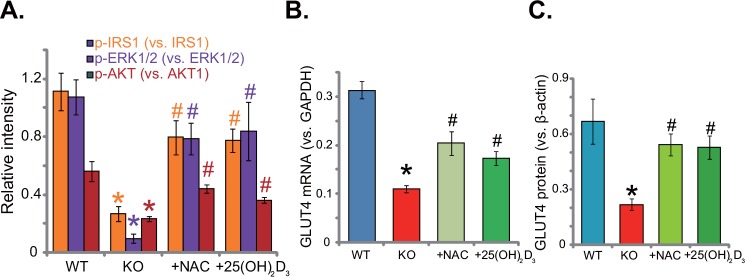
Supplement with NAC or 1,25(OH)2D3 rescues insulin signaling in 1α(OH)ase KO mice liver The liver tissues of wild-type (“WT”) mice, 1α(OH)ase knockout (“KO”) mice, or KO mice plus 1,25(OH)_2_D_3_ (thrice weekly subcutaneous injection), or NAC (in drinking water) were isolated; Expressions of listed proteins were tested by Western blotting assay (Results were quantified, **A** and **C**); *GLUT4 mRNA* expression was tested via the qRT-PCR assay **(B)**. Data were expressed as mean ± SE (n=5).* *p* <0.05 *vs*. “WT” mice. ^#^ *p* <0.05 *vs*. “KO” mice only.

This study aims to understand the link between vitamin D deficiency and insulin resistance. We thus analyzed insulin signaling in 1α(OH)ase knockout (“KO”) mice. Western blotting assay results (Quantified in Figure 2A) demonstrated that activation of insulin signalings, including IRS-1 (insulin receptor substrate-1), downstream ERK1/2 and AKT, was significantly lower in the liver tissues of KO mice, as compared to the WT mice. Remarkably, insulin signaling impairment in KO mice was almost completely inhibited with the supplement of the known anti-oxidant NAC (1 mg/mL) or 1,25(OH)_2_D_3_ (1 μg/kg) (Figure 2A). Further studies showed that glucose transporter 4 (GLUT4), a key glucose transporter protein, was also downregulated in the liver tissues of KO mice (Quantified in Figure 2B and 2C). Adding NAC or 1,25(OH)_2_D_3_ again restored*GLUT4 mRNA* (Figure 2B) and protein (Figure 2C) expression in KO mice livers. Since oxidative stress is crucial in the development of insulin resistance [6–8], we thus propose that oxidative stress could be a primary cause of insulin resistance in vitamin D-deficient mice.

### Supplement with NAC or 1,25(OH)_2_D_3_ alleviates oxidative stress in 1α(OH)ase KO mice liver

**Figure 3:**
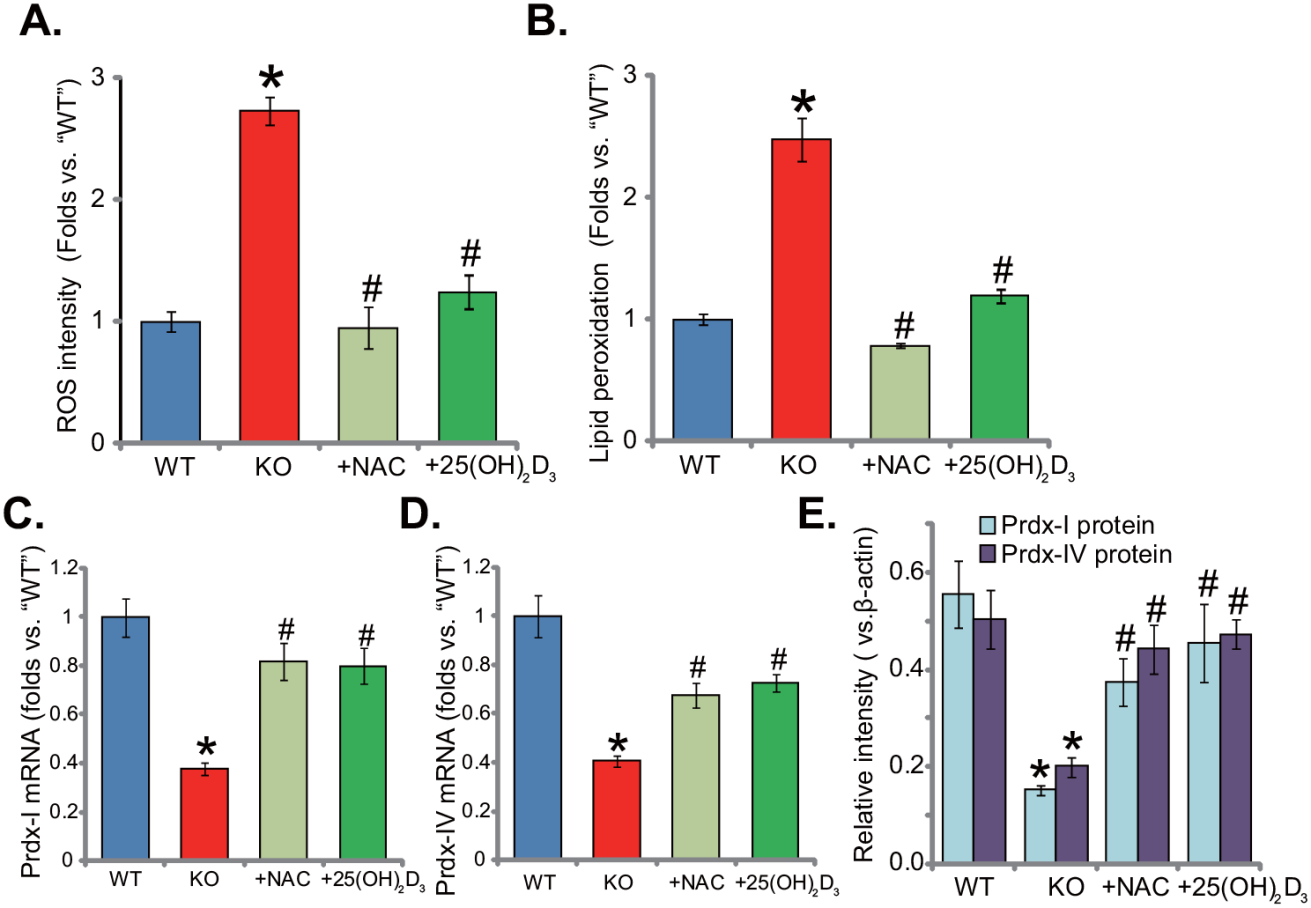
Supplement with NAC or 1,25(OH)2D3 alleviates oxidative stress in 1α(OH)ase KO mice liver The liver tissues of wild-type (“WT”) mice, 1α(OH)ase knockout (“KO”) mice, or KO mice plus 1,25(OH)_2_D_3_ (thrice weekly subcutaneous injection), or NAC (in drinking water) were isolated; Relative ROS level **(A)** and lipid peroxidation content **(B)** were analyzed; mRNA (**C** and **D**, qRT-PCR assay) and protein (Quantified in **E**, Western blotting assay) expression of Prdx-I and Prdx-IV were also examined. Data were expressed as mean ± SE (n=5). * *p* <0.05 *vs*. “WT” mice. ^#^*p* <0.05 *vs*. “KO” mice only.

Previous studies have demonstrated that both 1α(OH)ase and vitamin D3 are anti-oxidant [15–17]. Vitamin D binds to vitamin D receptor (VDR) to activate superoxide dismutase (SOD), phospholipid hydroperoxide glutathione peroxidase (GSH-Px) and possible other anti-oxidant enzymes [17]. On the other hand, vitamin D3 deficiency could cause oxidant stress [13]. Here, we showed that ROS content in the livers of KO mice was significantly increased (compared to the WT mice, Figure 3A). ROS intensity in KO mice was about three times higher than that of WT mice (Figure 3A). Meanwhile, lipid peroxidation intensity in the livers of KO mice was also increased (Figure 3B). Remarkably, adding known NAC or 1,25(OH)_2_D_3_ largely inhibited oxidative stress in KO mice (Figure 3A and 3B). Further studies showed that mRNA (Figure 3C and 3D) and protein (Quantified in Figure 3E) expression of two key anti-oxidative genes, peroxiredoxin I (Prdx-I) and peroxiredoxin IV (Prdx-IV), were both decreased in the livers of KO mice. Supplement with NAC or 1,25(OH)_2_D_3_ restored Prdx-I and Prdx-IV expression (Figure 3C-3E). Therefore, NAC or 1,25(OH)_2_D_3_ alleviates oxidative stress in KO mice liver.

### Supplement with NAC or 1,25(OH)_2_D_3_ alleviates p53-p21 signaling activation and apoptosis in 1α(OH)ase KO mice liver

**Figure 4:**
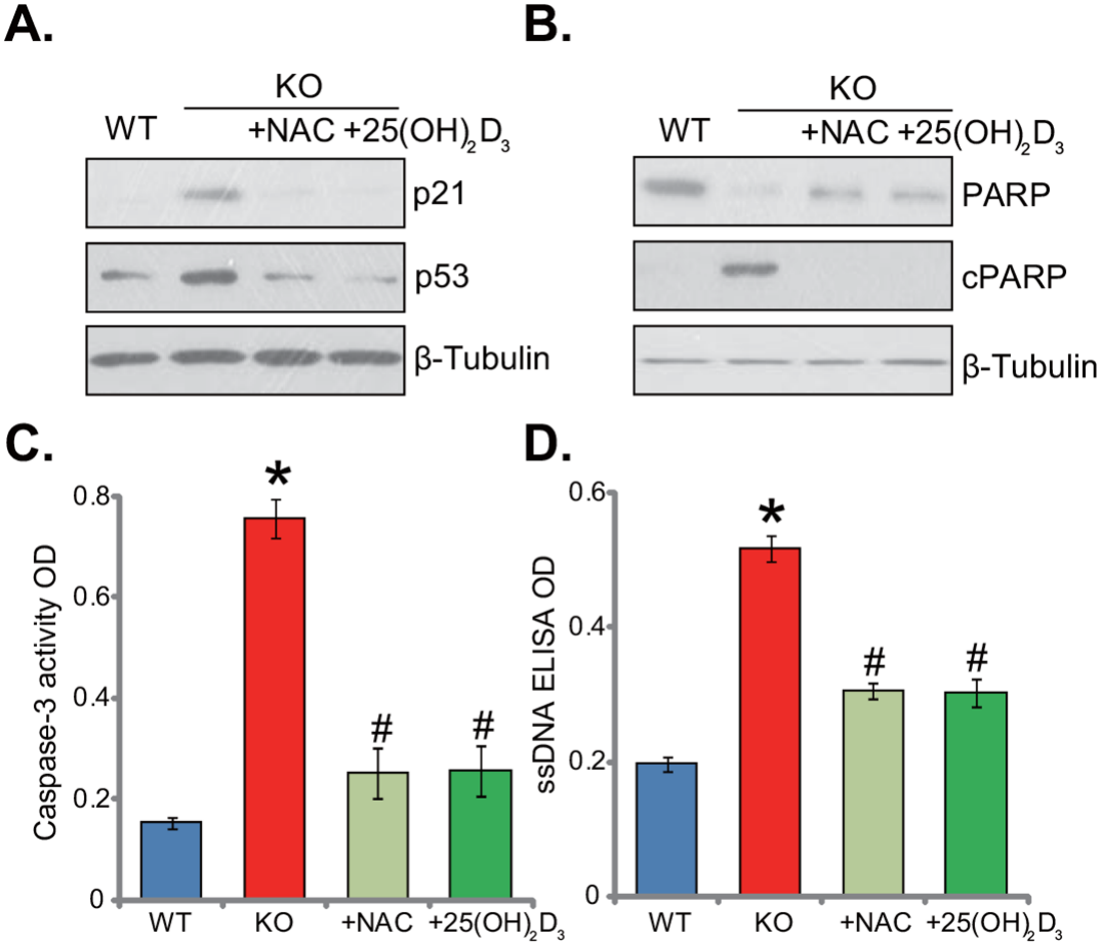
Supplement with NAC or 1,25(OH)2D3 alleviates p53-p21 signaling and apoptosis induction in 1α(OH)ase KO mice liver The liver tissues of wild-type (“WT”) mice, 1α(OH)ase knockout (“KO”) mice, or KO mice plus 1,25(OH)_2_D_3_ (thrice weekly subcutaneous injection), or NAC (in drinking water) were isolated; Expressions of listed proteins were tested by Western blotting assay **(A** and **B)**; Apoptosis was tested by the caspase-3 activity assay **(C)** and the ssDNA ELISA assay **(D)**. Data were expressed as mean ± SE (n=5). * *p* <0.05 *vs*. “WT” mice. ^#^ *p* <0.05 *vs*. “KO” mice only.

The results above demonstrated that 1α(OH)ase knockout induced oxidative stress in mice liver. It is known that oxidative stress could activate a number of signalings, including p53-p21 cascade [18, 19]. As shown in Figure 4A, expression level of p53-p21 was quite low in the livers of WT mice. Yet, p53 and p21 were both significantly upregulated in the KO mice livers (Figure 4A). Remarkably, supplement with NAC or 1,25(OH)_2_D_3_ largely inhibited p53 and p21 signaling in the KO mice livers (Figure 4A). ROS production and p53-p21 signaling activation might also induce cell apoptosis. Various assays were then applied to test apoptosis in the KO mice. As demonstrated, as compared to the WT mice, livers of KO mice presented with high level of PARP cleavage (Figure 4B), increased caspase-3 activity (Figure 4C) and augmented single strand DNA (ssDNA) ELISA OD (Figure 4D). These results indicated apoptosis activation in KO mice livers. Remarkably, supplement with NAC or 1,25(OH)_2_D_3_ largely attenuated apoptosis in livers of KO mice (Figure 4B-4D). Together, NAC or 1,25(OH)_2_D_3_ alleviates p53-p21 activation and apoptosis in KO mice livers.

### Supplement with NAC or 1,25(OH)_2_D_3_ attenuates inflammation in 1α(OH)ase KO mice liver

**Figure 5:**
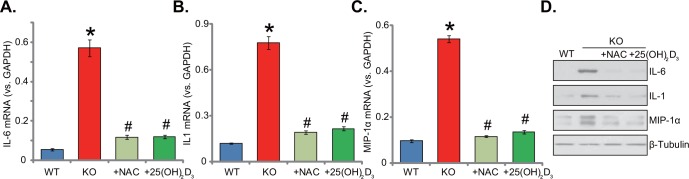
Supplement with NAC or 1,25(OH)2D3 attenuates inflammation in 1α(OH)ase KO mice liver The liver tissues of wild-type (“WT”) mice, 1α(OH)ase knockout (“KO”) mice, or KO mice plus 1,25(OH)_2_D_3_ (thrice weekly subcutaneous injection), or NAC (in drinking water) were isolated; mRNA expression of interleukin-6 (IL-6, **A**), interleukin-1 (IL-1, **B**) and macrophage inflammatory protein-1α (MIP-1α, **C**) were tested via the qRT-PCR assay; Protein expression of above cytokines was also shown **(D)**. Data were expressed as mean ± SE (n=5). * *p* <0.05 *vs*. “WT” mice. ^#^ *p* <0.05 *vs*. “KO” mice only.

Dysregulation of inflammation is another well-known contributor of insulin resistance [4, 20]. Previous studies have confirmed that vitamin D may increase the release of anti-inflammatory cytokines, whiling reducing many pro-inflammatory cytokines [20–22]. Here, we also showed that mRNA and protein expression of several key pro-inflammatory cytokines, including interleukin-6 (IL-6), interleukin-1 (IL-1) and macrophage inflammatory protein-1α (MIP-1α), was significantly increased in KO mice liver tissues (Figure 5A-5D). On the other hand, their expressions were relatively low in the liver tissues of WT mice (Figure 5A-5D). These results suggested that vitamin D deficiency possibly induced chronic inflammation in mice livers. Remarkably, supplement with NAC or 1,25(OH)_2_D_3_ largely attenuated chronic inflammation responses in the KO mice livers (Figure 5A-5D). Expression above pro-inflammatory cytokines (IL-6, IL-1 and MIP-1α) decreased almost to the level of WT mice after supplement with NAC or 1,25(OH)_2_D_3_ (Figure 5A-5D). These results implied that supplement with NAC or 1,25(OH)_2_D_3_ largely attenuated inflammation in the KO mice livers. Thus, ROS could be the primary cause of chronic inflammation in the KO mice.

### Supplement with NAC or 1,25(OH)_2_D_3_ reverses insulin resistance in 1α(OH)ase KO mice

**Figure 6:**
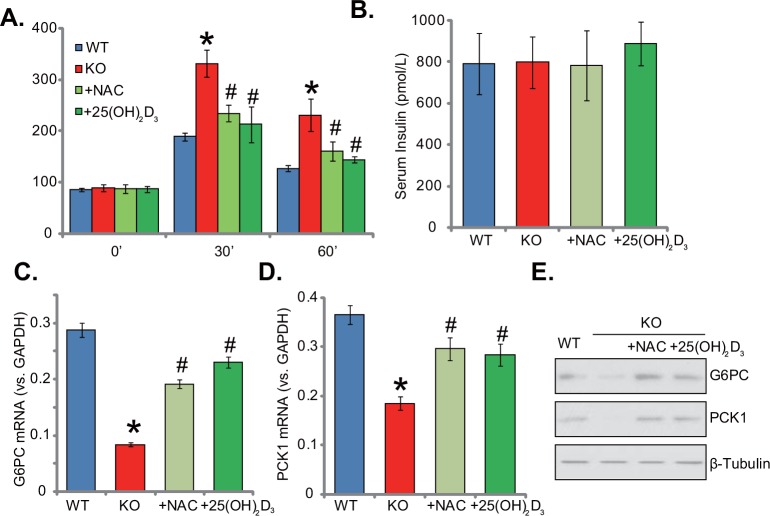
Supplement with NAC or 1,25(OH)2D3 reverses insulin resistance in 1α(OH)ase KO mice Wild-type (“WT”) mice, 1α(OH)ase knockout (“KO”) mice, or KO mice plus 1,25(OH)_2_D_3_ (thrice weekly subcutaneous injection), or NAC (in drinking water) were subjected to oral glucose tolerance test, and serum glucose level was tested **(A)**. Serum insulin level (at starvation time point) was also tested (**B**). mRNA (**C** and **D**, qRT-PCR assay) and protein (**E**, Western blotting assay) expressions of glucose-6-phosphatase-α (G6PC) and phosphoenolpyruvate carboxykinase (PCK1) in the liver tissues were presented. Data were expressed as mean ± SE (n=5). * *p* <0.05 *vs*. “WT” mice. ^#^ *p* <0.05 *vs*. “KO” mice only.

Thus far, we confirmed that insulin signaling was impaired in the 1α(OH)ase KO mice livers, where ROS and inflammation levels were both increased. These events should favor insulin resistance. Indeed, the oral glucose tolerance test (OGTT) results confirmed insulin resistance in the KO mice (Figure 6A). After initial glucose gavage, serum glucose level was significantly higher in KO mice than that in the WT mice (at 30-min and 60-min points, Figure 6A). Remarkably, glucose tolerance in the KO mice was largely attenuated with supplement of NAC or 1,25(OH)_2_D_3_ (Figure 6A). Notably, serum insulin level was not significantly different between the groups (Figure 6B). Glucose metabolism-related genes, including glucose-6-phosphatase-α (G6PC) and phosphoenolpyruvate carboxykinase (PCK1), were also tested. Results showed that mRNA (Figure 6C and 6D) and protein (Figure 6E) expressions of both genes were decreased in 1α(OH)ase KO mice livers, which were again largely attenuated with treatment of NAC or 1,25(OH)_2_D_3_ (Figure 6C-6E). Together, these results suggest that NAC or 1,25(OH)_2_D_3_ almost reverses insulin resistance in 1α(OH)ase KO mice.

## DISCUSSION

Vitamin D is extremely vital in maintaining the mineral homeostasis, regulating the balance of bone and calcium/phosphorus. Recent evidences have also implied a potential function of vitamin D deficiency in the development of insulin resistance and type II diabetes [4, 20, 23–25]. Low serum vitamin D level is often observed in a large proportion of patients with insulin resistance, glucose intolerance and obesity [4, 23, 24]. Meanwhile, an inverse link was established between serum vitamin D content and type II diabetes [4, 20]. In our study, insulin resistance was also developed in mice with 1α(OH)ase knockout, which is consistent with the previous findings [26, 27]. More importantly, exogenous supplementation of active vitamin D3 significantly alleviated insulin resistance in our study. Thus, vitamin D deficiency could be at least one important risk of insulin resistance [4, 20, 23–25].

1,25(OH)_2_D_3_ could function as an efficient anti-oxidant agent. Treatment with 1,25(OH)_2_D_3_ in several cell lines was shown to induce or activate anti-oxidant enzymes, including Prdx-I, Prdx-IV, thioredoxin reductase1 (TXNRD1), SOD, GSH-Px and others, which significantly limited oxidant stress [15–17, 28, 29]. On the other hand, 1,25(OH)_2_D_3_ deficiency could thus cause oxidative stress. The latter is a well-known contributor of insulin resistance [6, 7]. In the current study, we showed that the liver tissues in vitamin D-deficient mice also presented with impaired insulin signaling, decreased GLUT4 expression and increased ROS content. Meanwhile, p53-p21 signaling and apoptosis intensity were also elevated. Remarkably, such changes in the KO mice livers were largely attenuated by NAC treatment. The latter also dramatically attenuated insulin resistance and metabolic abnormalities in the KO mice. These *in vivo* evidences suggest that oxidative stress could be the primary cause of insulin resistance by vitamin D deficiency.

Recent studies [4, 21, 22], including ours [14], have also proposed an anti-inflammation function of vitamin D. Deficiency of vitamin D receptor (VDR) could result in severe inflammation in IL-10 KO mice [30]. On the other hand, a high serum vitamin D level often correlates with reduced risk for developing inflammatory diseases (*i.e*. inflammatory bowel disease) [31]. Meeker *et al*., showed that dietary vitamin D supplementation could prevent the production of pro-inflammatory cytokines [32]. In the current study, we show that KO mice probably also developed chronic inflammation in the liver. Expression of pro-inflammatory cytokines (IL-6, IL-1 and MIP-1α) were significantly increased in the KO mice livers. Remarkably, NAC treatment dramatically reduced the level of liver inflammation in the KO mice. Thus, ROS production is possibly a key trigger of chronic liver inflammation in the KO mice. The detailed mechanisms may warrant further studies.

## MATERIALS AND METHODS

### Animals

As previously described [13, 14], 1α(OH)ase knockout (“KO”) mice were generated through breed of heterozygous mice. The genotype of the mice was confirmed by PCR using mouse tail samples [13, 14]. Wild-type (“WT”) and KO mice were subjected to following regimens for three weeks: (1) WT mice only; (2) KO mice only; (3) KO mice plus thrice weekly subcutaneous injections of 1,25(OH)_2_D_3_ (1 μg/kg per mouse); (4) KO mice plus 1 mg/mL of NAC in drinking water daily. Following euthanasia, full liver was removed and washed in PBS, which was then frozen in liquid nitrogen before RNA and protein extraction. All mice were raised in the routine condition at the Soochow University's Animal Facility (Suzhou, China). All animal procedures were approved by Nanjing Medical University's Ethics Review Board and IACUC.

### Measurements of serum glucose, calcium, phosphorus and 1,25(OH)_2_D_3_

Before mice were sacrificed, serum was obtained, and serum calcium and phosphorus levels were examined via an auto-analyzer (Beckman Synchron 67; Beckman Instruments) [13, 14]. Serum 1,25(OH)_2_D_3_ content was tested with radioimmunoassay (DiagnosticProducts, Los Angeles, CA) [13, 14]. Serum insulin level was tested via a commercial available radioimmunoassay kit (GE, Shanghai, China).

### Oral glucose tolerance test

Mice were fasted overnight for 12 hours. Glucose (1 g/kg body weight) was administered through oral gavage. Blood was sampled from the tail vein at 0, 30 and 60 min. Serum glucose concentration was measured via an automatic glucose-meter (GE, Shanghai, China).

### Chemicals and reagents

NAC and 1,25(OH)_2_D_3_ were purchased from Sigma Aldrich Chemicals (Nanjing, China). The antibodies of this study were provided by Cell Signaling Technology (Danvers, MA) and Abcam (Shanghai, China).

### RNA isolation and qRT-PCR

As described in our previous studies [33, 34], RNA was isolated from frozen liver tissues with Trizol reagents (Invitrogen, Shanghai, China). Quantitative real-time PCR (“qRT-PCR”) reactions were performed via the SYBR green kit through the ABI-7600 PCR system (Applied Biosystems). mRNA primers of *1α(OH)ase* and GAPDH were described previously [35, 36]. mRNA primers for *IL-1*, *IL-6* and *MIP-1α* were described early [37]. mRNA primers for *G6PC* and *PCK1*, as well as *Prdx-I* and *Prdx-IV* were also reported early [38, 39]. The 2^ΔΔCt^ method was applied to calculate relative mRNA expression (vs. GAPDH mRNA). All the primers were synthesized by Genepharm (Suzhou, China).

### Western blotting assay

For each condition, 30-50 μg total lysate proteins per lane were separated by SDS-PAGE gels, which were transferred to the PVDF membranes. Afterwards, the blots were blocked, and were incubated with designated primary and secondary antibodies. Enhanced chemiluminescence (ECL) reagents (Pierce, Nantong, China) were applied to visual the targeted bands [40–42]. Total gray of each band was quantified through the ImageJ software, and the value was always normalized to the loading control (β-Tubulin/β-actin).

### Single strand DNA (ssDNA) ELISA assay of apoptosis

As described, ssDNA enzyme-linked immunosorbent assay (ELISA) kit was applied to test DNA fragmentation from 30 μg liver tissue lysates (per condition), using the commercial available photometric sandwich immunoassay of cytoplasmic ssDNA fragments (Roche, Shanghai, China).

### Caspase-3 activity assay of apoptosis

Liver tissue lysates (30 μg total lysates per condition) were tested via the Apo-ONE homogeneous caspase-3 activity kit (Promega, Shanghai, China), which determines the caspase-3 substrate via Rhodamine 110 fluorescence. Rhodamine 110 fluorescence intensity OD at 500 nm was recorded.

### Reactive oxygen species (ROS) assay

Liver tissues were homogenized, and homogenate (10%) was centrifuged at 4000 rpm at 4°C for 10 min. Supernatant was used for measurement of total superoxide dismutase (T-SOD) using the A001-1SOD detection kit from Nanjing Jiancheng Bioengineering Institute (Nanjing, China). All examinations were performed according to the manufacturer's instructions [14].

### Lipid peroxidation assay

As described [43, 44], the thiobarbituric acid reactive substances (TBAR) assay was applied to analyze the lipid peroxidation level in mice liver tissues. Briefly, liver tissue lysates (20 μg per condition) were incubated with 20% of acetic acid and thiobarbituric acid solution. After heating, the mixtures were centrifuged, and the red pigment dye in the supernatant was examined under a microplate reader. TBAR activity was expressed as nM of malondialdehyde per mg protein, and its value in treatment group were always normalized to that of WT mice control.

### Statistical analysis

The data were presented as the mean ± standard error (SE). Statistical differences were analyzed by one-way *ANOVA* followed by multiple comparisons performed with post hoc Bonferroni test (SPSS version 18.0). Values of *p* < 0.05 were considered as statistically significant.

## CONCLUSIONS

Based on the results, we conclude that inhibition of oxidative stress by NAC alleviated insulin resistance in vitamin D-deficient mice. Oxidative stress shall be the primary cause of insulin resistance by vitamin D deficiency.
